# Ayurvedic management of diabetic peripheral neuropathy: a case report

**DOI:** 10.3389/fmed.2026.1802855

**Published:** 2026-05-20

**Authors:** Arathy Menon, James Chacko, Delvin T. Robin, Preethi Mohan

**Affiliations:** 1 Department of Kayachikitsa, Shree Swaminarayan Ayurvedic College, Swaminarayan University, Gandhinagar, Gujarat, India; 2 Department of Kayachikitsa, Rajiv Gandhi Ayurveda Medical College, Mahe, Puducherry, India; 3 Department of Swasthavritta, Amrita School of Ayurveda, Amrita Vishwa Vidyapeetham, Kollam, Kerala, India; 4 Department of Agada Tantra and Vyavahara Ayurveda, Amrita School of Ayurveda, Amrita Vishwa Vidyapeetham, Kollam, Kerala, India

**Keywords:** ayurveda, diabetic peripheral neuropathy, type 2 diabetes mellitus, Panchakarma, neurodegeneration, case report

## Abstract

**Introduction:**

Diabetic neuropathy is a prevalent and debilitating complication of poorly controlled diabetes mellitus, primarily affecting peripheral nerves. Patients commonly present with symptoms such as burning sensation, numbness, tingling, and pain in the extremities, often associated with sensory deficits. Current conventional management provides limited symptomatic relief, highlighting the need for complementary approaches. In Ayurveda, this condition can be considered an *Upadrava* (complication) of *Prameha* (diabetes mellitus) and is managed through a combination of internal medications, external therapies, and detoxification procedures.

**Methodology:**

A 65-year-old male patient with a 15-year history of uncontrolled diabetes mellitus presented with a severe burning sensation in both lower limbs. The patient was managed with Ayurvedic interventions, including internal medications for 1 month followed by 21 days of inpatient Panchakarma therapy.

**Results:**

Clinical assessment using the visual analog scale (VAS), Neuropathy Disability Score (NDS), and neuropathy grading showed significant improvement following the intervention. The patient, who initially presented with moderate neuropathy, showed marked clinical improvement after 2 months of treatment.

**Conclusion:**

This case suggests that Ayurvedic interventions may offer a beneficial complementary approach in the management of diabetic peripheral neuropathy. Further systematic studies are required to substantiate these findings.

## Introduction

Diabetic peripheral neuropathy (DPN) is a common microvascular complication of type 2 diabetes mellitus, characterized by progressive damage to peripheral nerves. Chronic hyperglycemia contributes to long-term nerve injury, resulting in impaired sensory function. DPN most commonly affects the distal nerves of the lower limbs, particularly the feet. The prevalence of DPN increases with the duration of diabetes, with estimates in India ranging widely from 9.6 to 78% across different populations (1).

Diabetic neuropathy can be broadly classified into peripheral and autonomic types. Peripheral neuropathy, the most common form, is typically distal and symmetrical, initially affecting the feet and legs and later progressing to involve the hands and arms. Clinical manifestations vary depending on the nerves involved and are often more pronounced at night. Common symptoms include tingling, burning sensation, pricking, numbness, and heightened sensitivity to touch in the extremities. In advanced stages, patients may develop reduced perception of pain and temperature, along with impaired balance and coordination, leading to difficulty in walking on uneven surfaces. These deficits increase the risk of injuries, particularly in the lower limbs, which may progress to chronic, non-healing ulcers and subsequent infections. Muscle weakness may also be present in later stages of the disease.

In Ayurveda, diabetes mellitus can be correlated with *Madhumeha*, a subtype of *Prameha*. Although there is no direct description of diabetic peripheral neuropathy as a distinct entity, its clinical features may be understood in the context of *Poorvaroopa* (premonitory features) and *Upadrava* (complications) of *Prameha* (2, 3). Symptoms such as *Daha* (burning sensation) and *Suptata* (numbness) are described as premonitory features of *Prameha*. Based on clinical presentation, DPN may be interpreted as a condition predominantly involving Vata and Pitta dosha imbalance.

## Patient information

We report the case of a 65-year-old Indian man, a retired government employee, who presented with complaints of severe burning sensation and numbness in both lower limbs for the past 6 months, associated with unsteadiness while walking. He was a non-smoker and non-alcoholic.

The patient had a 15-year history of poorly controlled type 2 diabetes mellitus and was on insulin therapy (10 units daily) for the past 2 years. There was no significant family history of diabetes or neuropathic disorders, and no relevant psychosocial factors were reported.

Prior to presentation, the patient had not received any specific treatment for neuropathy. His symptoms had progressively worsened, affecting his daily activities and quality of life.

## Case report

Further clinical evaluation was performed to assess the severity of the condition. Laboratory investigations revealed a fasting blood sugar (FBS) level of 210 mg/dL, postprandial blood sugar (PPBS) of 298 mg/dL, and glycated hemoglobin (HbA1c) of 11.4%, indicating poor glycemic control.

The intensity of pain was assessed as 10/10 on the visual analog scale (VAS). The patient described the burning sensation as similar to “burning charcoal” placed over the legs.

There was no history of comorbid conditions such as hypertension, dyslipidemia, or thyroid disorders.

Based on the clinical presentation, the patient was initially managed with oral Ayurvedic medications, followed by a planned inpatient admission for 21 days for further therapeutic interventions.

## Examination

On general examination, the patient appeared anxious. The tongue was coated, appetite was adequate, and bowel and bladder habits were normal.

From an Ayurvedic perspective, the patient was in *Vridhavastha* (old age) with *Vata–Pitta Prakriti* (body constitution). He exhibited *Madhyama Samhanana* (moderate body build), *Madhyama Sara* (average tissue quality), *Sama Pramana* (proportionate body structure), *Madhyama Satmya* (moderate adaptability), *Madhyama Satva* (moderate मानसिक strength), *Madhyama Ahara Shakti* and *Jarana Shakti* (moderate food intake and digestive capacity), and *Avara Vyayama Shakti* (reduced physical endurance).

Vital parameters were within normal limits: blood pressure was 128/86 mmHg, pulse rate was 92 beats per minute, body weight was 71 kg, height was 170 cm, and body mass index (BMI) was 24.57 kg/m^2^.

Neurological examination revealed reduced sensation in the lower extremities in a glove-and-stocking distribution. Vibration sense, assessed using a 128-Hz tuning fork, was significantly reduced at the great toes bilaterally. Protective sensation, evaluated using a 10-g monofilament test, was reduced at multiple plantar sites. Pain sensation, assessed by the pinprick test, was decreased, and temperature sensation was also reduced over both feet.

Gait examination showed mild unsteadiness. Deep tendon reflexes were diminished in the lower limbs, particularly the ankle reflex. On local foot examination, there were no ulcers, fissures, callosities, deformities, or signs of infection. Peripheral pulses, including dorsalis pedis and posterior tibial pulses, were palpable bilaterally.

The detailed findings of sensory and reflex examination are summarized in Table 1. These findings were suggestive of severe peripheral neuropathy affecting the lower extremities.

**Table 1:** Neurological examination findings.

Test	Method	Finding
Vibration sensation	128-Hz tuning fork at great toe	Markedly reduced bilaterally
Protective sensation	10-g monofilament test at plantar sites	Loss of protective sensation at multiple plantar sites
Pain sensation	Pinprick test	Diminished in distal foot bilaterally
Temperature sensation	Warm/cold perception	Reduced over both feet
Deep tendon reflex	Ankle reflex	Diminished bilaterally

Laboratory investigations, including complete blood count, liver and kidney function tests, thyroid profile, vitamin D levels, and vitamin B12 levels, were within normal limits. However, vitamin B1 levels were found to be mildly reduced at baseline (62 nmol/L).

## Diagnosis and assessment

Based on clinical history, physical examination, and laboratory findings, the patient was diagnosed with diabetic peripheral neuropathy (DPN).

From an Ayurvedic perspective, the condition can be considered as *Prameha Upadrava* (complication of diabetes mellitus), particularly characterized by symptoms such as *Daha* (burning sensation), indicating involvement of *Vata–Pitta* imbalance.

The diagnosis of diabetic peripheral neuropathy (DPN) was made based on the patient’s long-standing history of uncontrolled diabetes mellitus, characteristic clinical symptoms, and objective findings on neurological examination. The presence of distal symmetrical sensory loss, reduced vibration perception, reduced reflexes, and abnormal scores on validated assessment tools such as the Diabetic Neuropathy Symptom Score (DNS) and Toronto Clinical Scoring System (TCSS) supported the diagnosis.

Differential diagnoses such as nutritional neuropathy (including vitamin B12 deficiency), drug-induced neuropathy, and other metabolic or neurological causes were considered. These were ruled out based on normal laboratory findings and the absence of relevant clinical history.

The chronicity of diabetes, poor glycemic control (HbA1c 11.4%), and the severity of symptoms indicated a moderate to severe stage of neuropathy, suggesting a guarded prognosis without appropriate intervention.

## Treatment protocol

Considering the chronicity of the disease, treatment was initiated at the outpatient level with Ayurvedic internal medications, including *Shatavari Chinnaruhadi Kashayam*, *Shiva Gulika*, *Dhanwantaram Ghritam*, *Abhra Bhasma*, and *Trivanga Bhasma*. After 1 month of therapy, the patient reported approximately a 25% reduction in burning sensation.

Subsequently, the patient was advised to undergo inpatient (IP) treatment. The initial phase included *Sarvanga Dhara* (therapeutic pouring of medicated liquid over the body) with *Dhanyamla* for 5 days, along with *Lepa* (topical application) over both lower limbs using *Guduchi Patradi Choorna* mixed with milk.

This was followed by *Snehapana* (internal oleation) with *Mahatiktaka Ghrita* for 6 days, administered in a classical escalating dosage pattern up to 170 mL on the final day, achieving *Samyak Snigdha Lakshanas* (adequate oleation).

After completion of *Snehapana*, the patient underwent *Sarvanga Abhyanga* (full body oil massage) using *Pinda Taila*, followed by a lukewarm water bath for 3 days. On the third day, *Virechana* (therapeutic purgation) was administered using *Avipatti Choornam* (25 g) with lukewarm water on an empty stomach. The patient achieved 13 purgation bouts (*Vegas*), corresponding to *Madhyama Shuddhi* (moderate purification), followed by 1 day of rest and *Samsarjana Krama* (post-procedure dietary regimen).

Following *Virechana*, the patient reported approximately a 75% reduction in burning sensation.

In the post-purification phase (*Shodhana*), internal medications including *Shatavari Chinnaruhadi Kashayam*, *Kaishora Guggulu*, *Dhanwantaram Ghritam*, *Abhra Bhasma*, and *Trivanga Bhasma* were administered. External application of *Khajita Pinda Taila* was also continued (Table 2).

**Table 2:** Timeline of internal medication.

Intervention	Dose	Anupana	Duration
Dhanwantaram Ghritam	5 mL twice daily after food	Morning—5 mg of Abhra Bhasma Evening—5 mg of Trivanga Bhasma	23/09/24 to 22/10/24 7/11/24 to 11/11/24
Shiva Gutika	Two tablets twice daily after food	Lukewarm water	23/09/24 to 22/10/24
Shatavari Chinnaruhadi Kashayam	20 mL of Kashaya twice daily before food	60 mL of lukewarm water	7/11/24 to 11/11/24
Kaisora Guggulu	Two tablets twice daily after food	Lukewarm water	7/11/24 to 11/11/24

Further inpatient management included *Sarvanga Abhyanga*, *Snana* (bath), and *Panchatikta Ksheera Basti* (medicated enema) for 5 days. After completion of 21 days of inpatient treatment (Table 3), the patient was discharged with near-complete relief of symptoms. The changes in blood glucose levels before and after treatment are summarized in Table 4.

**Table 3:** Timeline of external intervention.

Procedure	Medicine used	Duration
Sarvanga Dhara	Dhanyamla	23/10/24 to 27/10/24
Lepa over legs	Guduchi Patradi Choorna	23/10/24 to 27/10/24
Snehapana	Mahatiktaka Ghrita	28/10/24 to 2/11/24
Sarvanga Abhyanga + Snana	Pinda Taila + Lukewarm water	3/11/24 to 5/11/24
Virechana	Avipatti Choorna	5/11/24
Rest	Rest	6/11/24
Panchatikta Ksheera Basti	Panchatikta Ksheera	7/11/24 to 11/11/24
Abhyanga	Khajita Pinda Taila	7/11/24 to 11/11/24

**Table 4:** Glucose levels before and after treatment.

Test	Before treatment	After treatment	After 3 months of follow-up
FBS	210 mg/dL	112 mg/dL	98 mg/dL
PPBS	298 mg/dL	152 mg/dL	118 mg/dL
HbA1c	11.4%	8.2%	6.1%

During the course of treatment, the patient continued his prescribed allopathic medication for glycemic control under medical supervision.

Symptom assessment was performed before and after treatment using the Diabetic Neuropathy Symptom Score (DNS) (4) and the Toronto Clinical Scoring System (TCSS) (5).

## Results

The patient was assessed using the Diabetic Neuropathy Symptom Score (DNS) (Table 5) and the Toronto Clinical Scoring System (TCSS) (Table 6) before and after treatment. Improvement was observed across all assessment scales (Table 7) following 1 month of oral therapy and subsequent 21 days of inpatient treatment.

**Table 5:** Diabetic neuropathy symptom score.

Symptoms	Before treatment	After treatment	After 3 months of follow-up	Scoring
Unsteadiness on walking	1	0	0	1—present 0—absent
Numbness	1	0	0	
Burning and aching pain	1	0	0	
Pricking sensation	1	0	0	
Results	4	0	0	

**Table 6:** Toronto clinical scoring system.

Components	Before treatment	After treatment	After 3 months follow-up	Scoring
Symptom score				
Pain	1	0	0	1—present 0—absent
Numbness	1	0	0	
Tingling sensation	0	0	0	
Weakness	1	0	0	
Ataxia	1	0	0	
Upper limb symptoms	0	0	0	
Results	4	0	0	
Reflex				
Knee reflex—right	0	0	0	0—normal 1—reduced 2—absent
Knee reflex—left	0	0	0	
Ankle reflex—right	1	0	0	
Ankle reflex—left	1	0	0	
Results	2	0	0	
Sensory test score				
Pinprick	1	0	0	0—normal 1—abnormal
Temperature	1	0	0	
Light touch	1	0	0	
Vibration	1	0	0	
Results	4	0	0	

**Table 7:** Comparative outcome of assessment score.

Assessment scale	Before treatment	After treatment	After 3 months of follow-up
DNS	4	0	0
TCSS	10	0	0

On post-treatment evaluation, vitamin B1 levels improved to 89 nmol/L. The patient reported a marked reduction in symptoms, including burning sensation, unsteady gait, and weakness. Clinical examination also showed improvement in reflexes and sensory function. Additionally, the patient reported overall improvement in quality of life.

## Follow-up

The patient was followed up 3 months after completion of treatment to assess the sustainability of outcomes. At follow-up, significant improvement in glycemic parameters was observed. Fasting blood sugar (FBS) decreased from 210 mg/dL to 98 mg/dL, and postprandial blood sugar (PPBS) reduced from 298 mg/dL to 118 mg/dL. Glycated hemoglobin (HbA1c) improved from 11.4 to 8.2% post-treatment and further to 6.1% at 3 months.

Vitamin B1 levels showed progressive improvement from 62 nmol/L at baseline to 75 nmol/L post-treatment and 89 nmol/L at follow-up. In addition to biochemical improvement, the patient reported sustained symptomatic relief. Outcome assessment using DNS and TCSS also showed continued improvement.

## Safety assessment and adverse drug reactions

The patient was closely monitored throughout the treatment period for any adverse effects. On the first day of inpatient treatment, a mild burning sensation in the legs was noted following *Sarvanga Dhara* with *Dhanyamla*. This was managed with a local application of *Tiktaka Ghrita*, following which the symptom subsided.

No further adverse reactions were observed. The patient did not report any gastrointestinal discomfort, dermatological reactions, or systemic complications. Blood glucose levels were monitored regularly, and no significant fluctuations were noted.

During the course of *Snehapana*, the patient continued prescribed allopathic medication for glycemic control under medical supervision. Overall, the treatment was well tolerated, with no significant treatment-related complications.

## Economic evaluation of treatment

The treatment was conducted in a government healthcare setting; therefore, the patient did not incur any direct expenses. However, the estimated cost of similar treatment in a private healthcare setting is presented in Table 8. Actual costs may vary depending on institutional protocols and healthcare settings.

**Table 8:** Treatment cost analysis.

Treatment type	Duration	Approximate cost (in rupees)
Outpatient	1 month	1,500–2,000
Inpatient	21 days	~ 31,500 (~1,500 per day)

## Patient perspective

The patient reported that prior to treatment, the burning sensation in his legs was severe and persistent, significantly affecting his sleep and ability to perform daily activities. Following the course of treatment, he experienced marked relief in symptoms, improved walking stability, and better sleep quality. He reported that he was able to resume routine activities with greater ease and expressed satisfaction with the overall treatment experience.

## Discussion

Diabetic peripheral neuropathy is a chronic complication of diabetes mellitus associated with microvascular damage, oxidative stress, and chronic inflammation leading to progressive nerve dysfunction. In the present case, long-standing uncontrolled diabetes may have contributed to the development of neuropathy.

Although there is no direct reference to DPN in classical Ayurvedic texts, the condition can be understood as a *Prameha Upadrava*, with clinical features corresponding to *Vata–Pitta* predominant pathology. Symptoms such as *Daha* (burning sensation) and *Suptata* (numbness) support this interpretation.

The observed clinical improvement in this case may be attributed to a combination of mechanisms, including improved metabolic regulation, anti-inflammatory effects, and potential neuroprotective actions of the interventions. Ayurvedic therapies such as *Shodhana* (purification), *Snehana* (oleation), and *Basti* (medicated enema) may contribute to systemic detoxification, improved circulation, and restoration of physiological balance.

A limitation of this case is the absence of electrodiagnostic studies such as nerve conduction velocity (NCV) or electromyography (EMG), which are considered gold standard investigations for confirming diabetic peripheral neuropathy. The diagnosis in this case was based on clinical presentation and validated scoring systems.

Certain formulations used in this case, including *Dhanwantaram Ghritam*, *Shiva Gulika*, and *Kaishora Guggulu*, have been traditionally indicated in metabolic and neurological disorders. Ingredients such as *Shilajatu* are known for their antioxidant and adaptogenic properties, which may play a role in reducing oxidative stress and improving metabolic function.

The treatment started with Dhanwantaram Ghritam (6) (5 mL twice daily), combined with 5 mg of Abhra Bhasma (7) in the morning and 5 mg of Trivanga Bhasma (8) in the evening. Dhanwantaram Ghritam is Vatahara (alleviating Vata) and Rakta Prasadana (enhancing blood circulation). Trivanga Bhasma is Pittahara (alleviating Pitta) and is also antidiabetic in nature (9). Abhra Bhasma has shown efficacy in addressing hyperglycemia (10). Shiva Gutika (11), having Shilajatu as the main ingredient, is Pittahara in nature and is renowned for balancing the Tridoshas, enhancing immunity, and promoting Rasayana, which also contributes to the strengthening of Ojas (12). Moreover, Shilajatu is mentioned as Rasayana for alleviating the Upadrava of Prameha (13), and it exhibits potent free radical scavenger activity and has beneficial effects on the endocrine system. Numerous preclinical and clinical researches have illustrated the significant antidiabetic potential of Shilajatu (14).

On admission, Sarvangadhara using Dhanyamla, which is Sheeta (15) to touch but effectively restores digestive fire (Agni), aids in the removal of Ama, clears the circulatory channels, reduces excess fat, and improves circulation. Dhanyamla is especially beneficial in conditions dominated by Kapha and Vata doshas, helping to alleviate inflammation, helping in compacting obesity, and activating the nervous system (16). Dhanyamla helps in providing nutrients to nerve fibers that help reduce the signs and symptoms of DPN (17). In addition, Lepa with Guduchi Patradi Choorna was applied for its Pittahara properties.

Then, Snehapana with Mahatiktaka Ghrita (18) was administered for 6 days. Mahatiktaka Ghrita is particularly effective for Pitta-related disorders due to its Sheeta and Kleda Shoshana properties, which help to alleviate the primary condition as well as the associated complications.

Next, Sarvanga Abhyanga with Pinda Taila (19), followed by Ushna Jala Snana, was advised. Pinda Taila, as mentioned in the context of Vatarakta, helps to balance both Vata and Pitta doshas and reduce pain. Since Swedana is contraindicated (20) in Prameha, Ushna Jala Snana (a warm water bath) was used instead. Virechana with Avipatti Choorna was then performed. Virechana plays a crucial role in eliminating excess Kapha and cleansing the body of toxins while also reducing vitiated Pitta dosha (21). Avipatti Choorna further aids in balancing Pitta (22).

After Virechana, a day of rest was observed, followed by the Peyadikrama. Treatments were resumed after this phase. Khajita Pinda Taila was applied to both feet to alleviate the burning sensation (Daha). Then, Panchatikta Ksheera Basti was given for 5 days. Panchatikta Ksheera Basti, known for its Vata–Pitta balancing qualities, helped significantly reduce the patient’s symptoms (23).

Internally, Shatavari Chinnaruhadi Kashayam (24) was administered, which helped balance both Pitta and Vata doshas. Additionally, Kaisora Guggulu (25), indicated for Prameha, was prescribed for its Rasayana benefits, as well as its ability to balance Vata and Pitta and Raktaprasadana.

Thus, after a 21-day IPD treatment, the patient reported a remarkable 99% relief from his symptoms.

The sequential therapeutic approach involving internal medication, followed by Panchakarma procedures, may have contributed to both symptomatic relief and improvement in metabolic parameters.

However, as this is a single case report, the findings should be interpreted with caution.

### Strengths and limitations

The strength of this case lies in the comprehensive and integrative therapeutic approach, combining internal medications with Panchakarma procedures, along with objective assessment using validated clinical scoring systems such as DNS and TCSS. The observed improvement in both clinical symptoms and biochemical parameters, including glycemic control and vitamin B1 levels, adds to the clinical significance of the findings.

However, this report is limited by its single-case design, lack of a control group, and relatively short follow-up period of 3 months. Additionally, the absence of electrodiagnostic confirmation, such as NCV or EMG, limits objective validation of the diagnosis. The concurrent use of conventional antidiabetic therapy may also have contributed to the observed outcomes. Therefore, the findings should be interpreted with caution.

## Conclusion

The comprehensive Ayurvedic treatment protocol, comprising both internal and external therapies, was associated with significant improvement in the symptoms of diabetic peripheral neuropathy (DPN). A marked reduction was observed in the Diabetic Neuropathy Symptom Score (DNS) and Toronto Clinical Scoring System (TCSS) at the time of discharge.

The patient reported substantial relief from symptoms such as burning sensation, unsteady gait, and weakness, along with improvement in reflexes and sensory function. Additionally, the patient expressed overall satisfaction with the treatment outcomes.

This case suggests that Ayurvedic interventions may offer a beneficial complementary approach in the management of diabetic peripheral neuropathy. However, further controlled studies are required to validate these findings.

## Data Availability

The original contributions presented in the study are included in the article/supplementary material, further inquiries can be directed to the corresponding author.
